# The MLL/SET family and haematopoiesis^☆^

**DOI:** 10.1016/j.bbagrm.2020.194579

**Published:** 2020-08

**Authors:** Eric T.B. Antunes, Katrin Ottersbach

**Affiliations:** MRC Centre for Regenerative Medicine, University of Edinburgh, Edinburgh, Scotland, UK

## Abstract

As demonstrated through early work in Drosophila, members of the MLL/SET family play essential roles during embryonic development through their participation in large protein complexes that are central to epigenetic regulation of gene expression. One of its members, MLL1, has additionally received a lot of attention as it is a potent oncogenic driver in different types of leukaemia when aberrantly fused to a large variety of partners as a result of chromosomal translocations. Its exclusive association with cancers of the haematopoietic system has prompted a large number of investigations into the role of MLL/SET proteins in haematopoiesis, a summary of which was attempted in this review. Interestingly, MLL-rearranged leukaemias are particularly prominent in infant and paediatric leukaemia, which commonly initiate in utero. This, together with the known function of MLL/SET proteins in embryonic development, has focussed research efforts in recent years on understanding the role of this protein family in developmental haematopoiesis and how this may be subverted by MLL oncofusions in infant leukaemia. A detailed understanding of these prenatal events is essential for the development of new treatments that improve the survival specifically of this very young patient group.

## Highlights

• Members of the MLL/SET family of proteins are important epigenetic regulators during mammalian development.
• All members of the MLL/SET family are involved in regulating aspects of developmental and adult haematopoiesis.
• MLL1 is frequently involved in chromosomal translocations that initiate different types of leukaemia.

## 1 Introduction

**Fig. 1:**
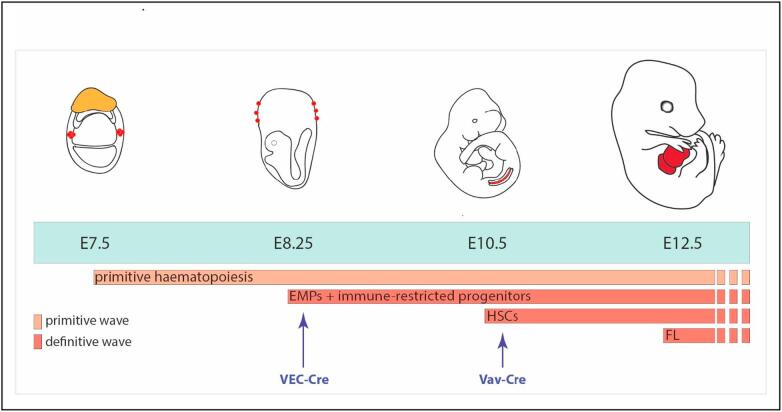
Developmental haematopoiesis. Depicted are key stages in the development of the haematopoietic system during mouse embryogenesis and how they can be differentially targeted with tissue-specific Cre recombinases. The first blood cells are generated in the E7.5 yolk sac in blood islands highlighted in red. A day later, the yolk sac gives rise to the first definitive haematopoietic progenitors, erythro-myeloid progenitors (EMPs) and their counterparts, immune-restricted progenitors. The first adult-type haematopoietic stem cells (HSCs) appear in the intra-embryonic aorta-gonads-mesonephros region, located between the forelimbs and hindlimbs of the E10.5 embryo as shown. HSC expansion and the establishment of an adult-type haematopoietic hierarchy occurs in the foetal liver (FL) from E12.5, thus making it the most important haematopoietic organ from midgestation.

Within a vertebrate embryo, blood development occurs at different anatomical sites through a series of transient events (reviewed in [1]). In mouse and before the onset of circulation, a primitive wave of megakaryocytes, macrophages and nucleated erythrocytes is generated in the extra-embryonic yolk sac starting from E7.5 (16–18.5 days post conception in human), and serves the initial oxygen and tissue requirements in the embryo [2,3]. A second extra-embryonic wave, definitive in nature, starting at E8.25 generates erythro-myeloid progenitors (EMPs) and immune-restricted progenitors within a functional vascular system [4,5]. The definitive route to a functional adult blood system is initiated at E10.5 within the intra-embryonic aorta-gonads-mesonephros (AGM) region [[6], [7], [8]]. This generates the first multipotent haematopoietic stem cells (HSCs) that are defined by their capacity to self-renew and provide multilineage haematopoietic reconstitution upon direct transplantation. Following this, blood progenitors circulate and seed the foetal liver from E11, where the system proliferates and differentiates further to establish an adult-type haematopoietic hierarchy apparent from E12.5 (Fig. 1), before colonising the bone marrow from E18, as the main haematopoietic site throughout adulthood [1].

For haematopoiesis, HSCs must differentiate and enter the cell cycle to begin the production of the blood lineages through the sequential generation and lineage-restriction of intermediate progenitor cells that possess multipotent and unipotent capacities. This hierarchical range of lineage potency allows for the dynamic usage of HSCs and multipotent progenitors (MPPs) for the maintenance and regeneration of blood during native or stressed conditions. Although far from complete, single-cell and lineage-tracing technologies are increasingly resolving the cellular heterogeneity within the classically defined haematopoietic stem and progenitor (HSPC) populations. The view that haematopoiesis occurs within an absolute hierarchical framework governed by discrete transitions through increasingly restricted progenitors is being challenged, towards a framework where there is a continuous landscape of differentiation towards the terminal lineages that emerges from transitionary low-primed HSPC states (reviewed in [9]).

Given the requirement for dynamic functionality, chromatin is suitably poised as an amenable platform to dictate blood function. At the nucleosome – the basic subunit of chromatin – approximately 146 base pairs of double-stranded DNA is wrapped around an octamer of histone proteins. Given that nucleosomes are innately inhibitory to transcription, nucleosomal modification or displacement is required to allow for gene expression [10,11]. This can be achieved through the action of multi-domain proteins that respond to intrinsic and extrinsic cellular signals to modify histones or assemble in multi-protein complexes and configure chromatin interactions and structure, thereby facilitating cell-specific gene expression.

In mammals, a family of six methyltransferases that can mono-, di- or tri-methylate histone 3 on lysine 4 (H3K4) through a highly conserved catalytic Su(var)3–9, Ezh2, Trithorax (SET) domain have become notable for their role in blood function, cancer and development [[12], [13], [14]]. Known as the mixed-lineage leukaemia (MLL)/SET or COMPASS family, the shared catalytic domain was first purified and characterised as a multi-protein complex in yeast *S. cerevisiae* as SET1, where it is entirely responsible for H3K4 methylation [[15], [16], [17], [18], [19]]. This singular yeast enzyme shares homology with three proteins in *D. melanogaster* known as SET1, Trithorax (Trx) and Trithorax-related (Trr), also responsible for H3K4 methylation [[20], [21], [22], [23], [24], [25]]. Seminal work in the fly showed that Trx, a part of the Trithorax protein group (TrxG), maintains initiated patterns of HOX gene expression that function to specify body segment identity [20,[25], [26], [27]]. TrxG antagonise the repressive action of the Polycomb protein group (PcG), allowing both systems to control these transcriptional states over successive cell divisions during morphogenesis [20,28,29].

The six MLL/SET paralogs in vertebrates likely reflect a historical duplication event of ancestral SET genes [30]. The family can be split into pairs: MLL1/MLL2 (KMT2A/B), MLL3/MLL4 (KMT2C/D) and SETD1A/SETD1B (KMT2F/G) based on sequence conservation, which suggests that SET containing proteins have diverged to acquire further and distinct functionalities beyond enzymatic function [14]. Much has been well documented about the history of discovery, structure and distinct functions of *S. cerevisiae* SET1, the *D. melanogaster* SET family and the mammalian MLL/SET proteins in development and disease [13,14,20,30]. This review will aim to describe how the MLL/SET family contribute to haematopoiesis, whilst drawing relevant information from studies of MLL1-associated leukaemia and development, where much work has been conducted.

## 2 A short guide to the structures and interactions of the MLL/SET family

The MLL/SET family commonly share a minimal core complex required for proper histone methyltransferase (HMT) activity that consists of the WD-40 repeat-containing protein 5 (WRD5), Retinoblastoma-binding protein 5 (RBBP5), absent, small, or homeotic-like (Drosophila) (ASH2L) and Dumpy-30 (DPY30) (WRAD) [[31], [32], [33], [34], [35]]. By building on the repertoire of possible binding modes that reconstituted mammalian MLL/SET and yeast COMPASS utilise when binding the nucleosome, high-resolution structural data are beginning to demonstrate how the WRAD scaffold functions to coordinate the activation of the SET domain [[36], [37], [38], [39], [40]]. Nucleosomal specificity and catalytic activation is mediated through an initial contact with a monoubiquitinated histone at lysine 120 (H2BK120ub1) which sets in motion a cascade of events within the catalytic module and towards the nucleosome, that allosterically prime the SET domain to sequester the N-terminal tail of H3 for subsequent methylation [36,[40], [41], [42]]. Substitution of a series of residues from MLL1 that connects WRAD to the MLL1 SET domain markedly increased MLL3 activity, by allowing the MLL3 chimera to assume a catalytic conformation more like MLL1, which negated the repressive action of WRD5 usually seen in the native MLL3 complex [36]. The catalytic efficiency of both mammalian MLL1/3 and yeast COMPASS are markedly reduced when binding an unmodified nucleosome [36,41]. Thus, H2BK120ub1 indirectly simulates enzymatic activity, and differences in inter-subunit structure contribute to the varying affinities within the MLL/SET family for HMT potential.

**Fig. 2:**
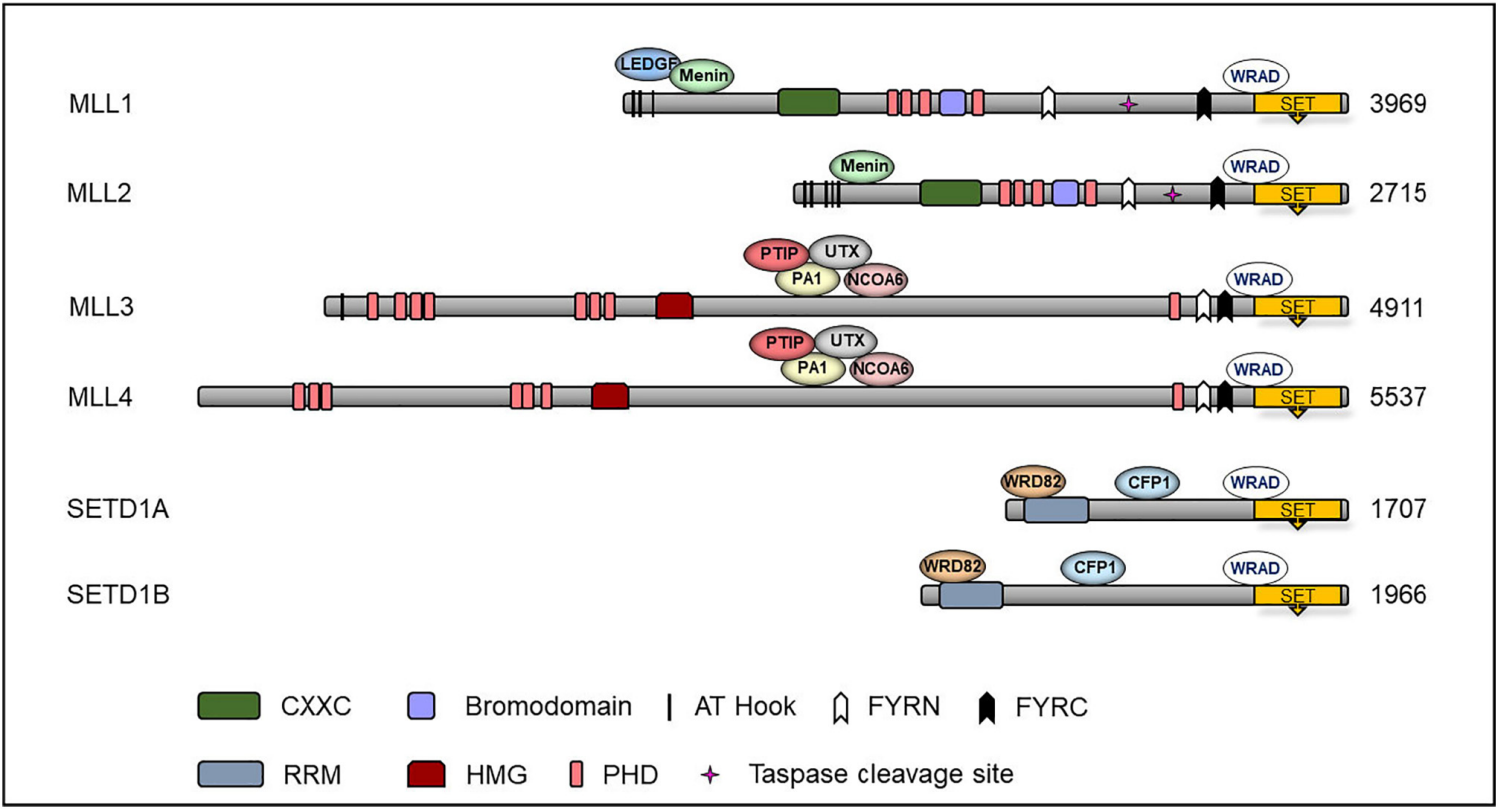
MLL/SET family domain structure and interactions. This representation shows the basic domain structure and protein interactome for all six MLL/SET family proteins. At the C-terminus, all commonly share an interaction with the WRAD scaffold that coordinates the activation of the catalytic SET domain, responsible for H3K4 methylation. Different domains between and within MLL/SET pairs are responsible for protein and DNA interactions. The numbers indicate the length in amino acids of the protein sequences. AT-hooks, adenosine-thymidine-hook; CXXC, Zinc finger-CXXC domain; FYRN/C, phenylalanine and tyrosine rich region (N- and C-terminal); HMG, high mobility group; RRM, RNA recognition motive; PHD, plant homeodomain; SET, Su(var)3–9, Ezh2, Trithorax; CFP1, CXXC finger protein 1; LEDGF, lens epithelium-derived growth factor; NCOA6, Nuclear Receptor Coactivator 6; PA1, PAXIP1-associated glutamate-rich protein 1; PTIP, PAX transcription activation domain interacting protein; NCOA6, Nuclear receptor coactivator 6; UTX, lysine-specific demethylase 6A; WDR82, WD repeat-containing protein 82; WRAD, WD-40 repeat-containing protein 5, RBB5, Retinoblastoma-binding protein5, ASH2L (Absent, Small, Or Homeotic)-Like (Drosophila) and DPY30, Dumpy-30.

Whilst they all share a relatively small SET domain, all MLL/SET members contain unique combinations of structural domains, with domain usage being closely shared between their respective pairs (Fig. 2). The WRAD complex also serves as a platform for the recruitment of interaction partners that further multi-domain functionality. Trx-like MLL1 and MLL2 are both cleaved by Taspase 1 [43]. For MLL1, this generates 320 kDa N-terminal and 180 kDa C-terminal fragments, which non-covalently dimerise via FYRN and FYRC domains to form the first hub for multi-protein complex assembly [[44], [45], [46], [47]]. Both retain all chromatin and protein interaction functionality at the N-terminal fragment, and catalytic SET function at the C-terminus (Fig. 2). Further contributors to MLL1 and MLL2 function are unique interactions with menin and, specific to MLL1, the formation of a ternary complex with the lens epithelium-derived growth factor (LEDGF) [[48], [49], [50], [51], [52], [53]]. Furthermore, MLL1 co-localises with transcriptionally activating lysine acetyltransferases and PAF1, indicating an ability to associate with different factors to target specific functions [[54], [55], [56], [57], [58]]. Thus far, only MLL2 interacts with AKAP95 [59]. Trr-like MLL3 and MLL4 do not bind to menin or LEDGF, but specifically interact with p53, UTX, PA1, PTIP and NCOA6 (Fig. 2) [[60], [61], [62], [63], [64]]. Structurally, both SETD1A and SETD1B are highly similar and share the interaction partners CFP1 and WDR82 (Fig. 2) [65,66]. Both lack a CXXC domain found in MLL1 and MLL2, and exploit its presence in CFP1 to target activity to gene promoters [67,68]. WDR82 forms a link to the C-terminal phosphorylated serine 5 on RNA polymerase II at actively transcribing genes [69,70]. For further reading there is extensive and excellent literature on the full spectrum of shared and unique interacting partners for MLL/SET proteins [14,71].

## 3 MLL1 is required for foetal and adult blood

The first indication that MLL1 may be required in haematopoiesis came from its identification in chromosomal translocations of intractable mixed-lineage infant leukaemias. Subsequent sequencing by independent groups revealed that MLL1 was functionally orthologous to Trx in *Drosophila* [27,[72], [73], [74]]. Given that it was known that Trx functions as a homeotic gene regulator during embryonic development, this set the framework for a series of mouse germline knockout experiments aimed at understanding how Mll1 functions in mammalian cells.

All Mll1 mutants presented with shifted anterior-posterior body axes, owing to Mll1 failing to maintain initiated Hox gene expression patterns from E8.5 [75,76]. This showed that Mll1 is essential for proper axial skeleton formation in the embryo and confirmed the functional conservation between Mll1 and Trx. Haematopoietic defects were most prominent in null embryos, which were lethal from E10.5 to E16.5 owing to differences in gene targeting [[76], [77], [78], [79]]. Homozygous mutants were characterised by reduced cellularity in the foetal liver and reduced myeloid colony forming units (CFU) derived from either the yolk sac at E10.5 or foetal liver at E12.5. *Mll1*^−/−^ HSPCs still differentiate but later as compared to wild-type cells, with no abnormalities in lineage distribution across all genotypes. Thus, the reduction in total output and delayed onset of proliferation indicates that Mll1 acts to mediate the generation of HSPCs and not maturation. Gene expression analyses showed that Mll1 loss is linked to reduced expression of Hoxa7/8/10 in the foetal liver [78]. Despite HSPC loss in *Mll1*^*−/−*^ embryos, those that do remain can support an expansion of Ter119+ erythroid cells between E13.5-E14.5 and, phenotypically, the embryos appear to be able to support erythropoiesis [79]. In contrast, heterozygosity causes a mild anaemia and shifts HOX gene expression posteriorly as compared to total loss in *Mll1*^*−/−*^. Haploinsufficient embryos also present with anaemia, thrombocytopenia and reduced numbers of B-lymphocytes and CFU, indicating that MLL gene dosage is critical to blood development and may also be relevant in MLL1-rearranged leukaemia, as only one wild-type allele remains when the other one is involved in a chromosomal translocation [[76], [77], [78]]. In line with previous results, Mll1 null embryonic stem cells (ESCs) are unable to generate lymphoid or myeloid progeny in the foetal liver of chimeric mice [80]. Injection of dissociated E11.5 whole AGM tissue into sublethally irradiated recipients revealed an absence of donor contribution from *Mll1*^−/−^ embryos, and very low-level reconstitution from heterozygotes, confirming that Mll1 is required for the first functional HSCs [80]. These findings showed that Mll1 is essential for the specification of functional HSPCs during embryogenesis and that haploinsufficiency has a pathological role.

As opposed to global deletion, blood-specific inducible knockout systems of Mll1 deletion were developed and revealed a loss of severity in observed phenotype, likely as a consequence of losing intrinsic Mll1 function alone [79,81,82]. During foetal development, constitutive haematopoietic-specific Mll1 deletion with Vav-Cre showed no changes in the HSPC numbers between E13.5 - E15.5 or differences in blood lineage distribution, which contrasts with the germline knockouts [79,82]. Yet, *Mll1*^−/−^ foetal HSPCs survive to birth, which is shortly followed by catastrophic bone marrow failure within 3 weeks [82]. Indeed, Vav-Cre-deleted *Mll1*^−/−^ foetal HSPCs have functionally defective CFU output and are unable to reconstitute irradiated adult recipients in competitive transplants. Therefore, during embryonic development, Mll1 is intrinsically required for the specification of transplantable HSCs and plays a part in HSPC differentiation (Table 1). The loss of severity in the conditional knockouts could result from external support mechanisms that do not function in the germline mutants.

**Table 1:** MLL/SET family function in foetal haematopoiesis.

	Foetal haematopoiesis							
Gene	Required for definitive HSC specification?	Mediate stressed HSC proliferative response?	Maintain steady-state progenitor numbers?	Median Vav-cre latency from birth	Progenitor expansion after transplant?	Required for HSPC expansion?	Methyltransferase activity required for haematopoiesis?	HSPC transcriptional response following deletion
MLL1	Vav-Cre No change in ST-HSC or LT-HSC numbers at E13.5 in FL [79] No change in LSK CD48^−^ numbers by E15.5 in FL [82]	Very low short term and no long term repopulation capacity [82]	No change in LSK CD48^−^ numbers by E15.5 in FL (Vav-Cre) [82]	3 weeks [82]	Not quantified	Significantly defective myeloid output in CFU [79,82]	No, foetal haematopoiesis proceeds normally (Vav-Cre); no change in global H3K4 [84] In ESC, promotes mesodermal and haematopoietic cell fate [113]	Loss of HoxA cluster in HSPC [76,78,79,80] HoxB cluster in ESC [125]
MLL2	Germline KO only – No indication for requirement in haematopoiesis [88]	No analysis	No analysis	No analysis	No analysis	No analysis	No SET mutants No ChiP in null HSPCs	No analysis
MLL3	Germline KO mice die shortly after birth, no obvious defects [94] Note - All experimental data from p53 null E13.5.-E14.5 FL HSPCs transplanted into adult mice [102]	shRNA - competitive transplants – increased number of LT-HSC, loss of ST-HSC [102]	No analysis	No Vav-Cre mice	Reduced MPP, CMP, GMP, no change to MEP [102]	Reduced myeloid CFU output [102]	No SET mutants in blood context Loss of H3K3me3 at some observed loci [102]	Downregulation of early haematopoietic progenitor differentiation, correlation to 7q MDS [102]
MLL4	Germline KO mice die early at E9.5 [94]	–	–	No Vav-Cre mice	–	–	Akin to germline knockout, SET mutants are embryonically lethal by E9.5, with no obvious abnormalities [99]	N/A
SETD1A	Vav-Cre – No LSK 9 days post birth suggestive of requirement during emergence of definitive HSCs [111]	No LSK generated [111]	No LSK generated [111]	7–20 days [111]	No LSK generated [111]	No LSK generated [111]	In ESC, promotes mesodermal and haematopoietic cell fate [113,114]	N/A
SETD1B	Vav-Cre - Haematopoiesis proceeds normally [112]	Not analysed in foetal cells	Not analysed in foetal cells	25 weeks [112]	Not analysed in foetal cells	No test of null foetal HSPCs in CFU	No SET mutant No ChiP in foetal HSPCs	No analysis

During adult haematopoiesis, acute Mx1-Cre Mll1 deletion following polyinosinic:polycytidylic acid administration leads to rapid bone marrow failure within 3 weeks as demonstrated by an overall loss in cellularity and widespread depletion of the HSPC compartments [81]. Less penetrant *Mll1* alleles in Vav-Cre mice showed that the B-cell and not T-cell development was especially susceptible to the loss of Mll1. All models demonstrated a specific loss in total number of HSPCs, defective CFU output in both myeloid and lymphoid conditions, and a total loss of engraftment capacity [79,81,82]. Mechanistically, Mll1 loss caused a failure to maintain quiescence in HSCs, which depleted the pool. Excision of *Mll1* via retroviral induction of Cre in adult myeloid-erythroid progenitors resulted in a two-fold reduction in CFU. Furthermore, mutant myeloid-erythroid progenitors were not able to re-enter the cell cycle following the re-addition of cytokines. Therefore, Mll1 is required during adult haematopoiesis to maintain functional HSCs through quiescence and it sustains cytokine-mediated proliferation of at least myeloid-erythroid progenitors.

### 3.1 Mll1 and B-cell development

Initially, there were conflicting reports for the requirement of Mll1 in the B-cell compartment. Vav-Cre-deleted *Mll1*^−/−^ mice displayed reductions in the numbers of B-cells, much more than T-cells [82]. However, as bone marrow failure was significant, this may have impaired B-cell output more than T-cell output. Contrastingly, CD19-Cre mediated *Mll1* deletion in adult mice showed no change in B-cell numbers [81]. To circumvent the previously lethal bone marrow phenotype, Rag1-Cre (targeting B and T lymphocytes) mice were crossed with *Mll1* floxed animals, which mediates deletion from late gestation to adulthood [83]. Significant losses in B-cell numbers were seen in both foetal and adult mice, in the bone marrow, spleen, blood and lymph nodes and highest between 2 and 3 weeks. T-cell numbers remained unaffected. Mll1 deficiency impaired B-cell differentiation at the pre-BCR checkpoint, thus blocking the transition from pro-B to pre-B resulting in reduced survival. This was attributed to attenuated RAS-MEK-ERK signalling downstream of the pre-BCR causing reduced ERK1/2 phosphorylation. Further implicated was a network of various mRNAs and microRNAs, and no single candidate gene could explain the observed phenotype, suggesting that these RNA candidates act between Mll1 and RAS-MEK-ERK signalling. Further insight into Mll1 function during normal B-cell development has been derived from the analysis of MLL-AF4 patient data, where an increased MLL1 and BCL6 expression signature was identified [84]. Virally induced Cre-mediated deletion of Mll1 in murine pre-B and mature splenic B-cells resulted in a loss of Bcl6 upregulation from 2 weeks. Interestingly, doxycycline-mediated induction of Bcl6 expression in murine pre-B cells increased Mll1 expression through an indirect mechanism, where Bcl6 represses the expression of Bmi1, Ctbp2 and Kdm2B, which in turn are transcriptional repressors of Mll1. This suggests that Mll1 and Bcl6 are connected through a positive feedback loop, which appears to be conserved in leukaemic cells [84].

### 3.2 Mechanisms of MLL1 function

To understand how Mll1 operates in cells, the effect of deleting its catalytic SET domain was explored. Homozygous deletion of the Mll1 SET domain reduces H3K4me at Hox loci resulting in their misexpression [85]. Despite this, mice survive embryogenesis through to adulthood with only mild skeletal defects suggesting non-SET dependant functionality. Mishra et al. utilised this model in HSPCs to demonstrate that the loss of HMT activity is compatible with normal haematopoiesis in adult mice and is dispensable for MLL-AF9-mediated transformation, one of the most common MLL1 leukaemogenic oncofusions. The loss of SET or even Mll1 in its entirety did not affect global H3K4me levels, suggesting this histone modification is not required to maintain Mll1 target gene expression. Instead, Mll1 recruits the acetyltransferase MOF for H4K16ac at the transcription start site (TSS) for this purpose [54,86]. Inhibition of Sirt1, which removes H4K16Ac, recused the loss of transcription following *Mll1* deletion at Mll1 target genes, indicating that it opposes MOF activity.

In adult mice, microarray analysis of CD48- Lin- Sca1+ Kit+ (LSK) HSPCs 6 days following Mx1-Cre-mediated excision showed that Mll1 operates within a molecular network that extends beyond the regulation of Hox genes [87]. Following deletion using both Mx1-Cre and estrogen receptor (ER) Cre inducible knockout models, five genes, *Mecom, Prm16, Hoxa9, Pbx1 and Eya1*, were identified as consistently downregulated and found to have Mll1 bound at their TSS. Overexpression of these targets in wild-type and *Mll1*^−/−^ LSK cells showed that no individual protein can restore normal expression levels of the proposed network in *Mll1*^−/−^ cells, with the exception of Evi1 which increased Prdm16 and Hoxa9, thus, these proteins are likely to function independently as downstream effectors of Mll1 function. Transplantation of *Mll1*^−/−^ cells that overexpress Prdm16 and Hoxa9 showed that these direct Mll1 targets are the most capable at rescuing the loss of HSC function by restraining proliferation. These results showed that Mll1 does not require its intrinsic methyltransferase activity to perform its functions, and that MLL1 fusions can operate through distinct mechanisms.

## 4 MLL2 in haematopoiesis

Despite significant homology, germline Mll2 deletion does not phenocopy what is seen with Mll1. In *Mll2*^−/−^ embryos, earlier growth retardation becomes increasingly apparent from E6.5 with no obvious cell type-specific defects by E9.5, and the embryos die by E10.5 through widespread apoptosis [88]. The maintenance of the mesodermal marker Mox1 and Hoxb1 was dependant on Mll2. Indeed other HoxB cluster genes were deregulated, which differs from Mll1, which mediates the HoxA and HoxC clusters. After E10.5, Rosa26-CreERT2-mediated *Mll2* loss had no effect on embryogenesis, including blood development; however, Mll2 does play a non-redundant role in oocytes as the main H3K4me2/3 HMT and in male and female fertility [89,90].

During adulthood, the use of a Rosa-CreERT2 model showed that Mll2 is required to mediate proper cytokine signalling during macrophage function [91]. Following lipopolysaccharide (LPS) stimulation, *Mll2*^−/−^ macrophages display attenuated intracellular NF-κB signalling because of reduced Tlr4 activation. This was a direct consequence of Pigp loss, which functions to add glycophosphatidylinositol to transmembrane proteins. This caused a loss of CD14 anchoring at the cellular membrane, which functions with Tlr4 in response to LPS. At the *Pigp* gene promoter and other direct Mll2 targets, deletion led to large increases in H3K27me3 suggestive of a requirement for H3K4me3 in opposing PcG repression. Alternatively, although some did, the majority of promoters that lost H3K4me3 experienced no changes in expression, suggesting that there are loci-dependent rules that dictate the relationship between Mll2 HMT or transcriptional effector activity and function.

## 5 MLL3 and MLL4 in haematopoiesis and blood disease

MLL3 and MLL4 are predominantly associated with the deposition of H3K4me1 at enhancers [[92], [93], [94]].This functionality is consistent with the sole Drosophila homolog Trr [24,95]. Trr loss does not result in embryonic HOX gene misexpression as is seen with Trx (homolog of MLL1/2), nor can Trr mutants influence Trx or PcG mutant homeotic phenotypes [22]. Both MLL3 and MLL4 function to prime enhancers as coactivators with the H3K27 acetyltransferases CREB-binding protein (CBP)/p300 which are hallmarks of active enhancers, a function that is likely independent of the SET domain [75,94,[96], [97], [98]]. Individually, Mll3-null mice die shortly after birth with no obvious morphological defects, whilst Mll4-null embryos show lethality at approximately E9.5 [94]. Corresponding to a requirement for HMT activity during embryogenesis, Mll4 SET deletion causes lethality at approximately E10.5 [99]. Both perform partially redundant functions during adipogenesis and myogenesis with Mll4 acting as the major H3K4me1/2 methyltransferase [94,100,101].

In the context of leukaemia, it is known that MLL3 functions as a tumour suppressor in 7q acute myeloid leukaemia (AML), and short hairpin RNA (shRNA) knockdown or CRISPR-Cas9-mediated deletion of Mll3 generates a transplantable and fatal leukaemia in p53-null HSPCs [102]. Competitive transplantation of p53-null shMLL3 HSPC increases the frequency and number of LT-HSC, whilst decreasing downstream multipotent progenitors (MPP), suggesting a role for Mll3 in mediating differentiation from HSCs [102]. Concordantly, transplanted mice show reduced white blood cell and platelet counts and a decreased contribution from shMll3-targeted HSPC to common myeloid progenitor (CMP) and granulocyte-monocyte progenitor (GMP), although not megakaryocyte-erythrocyte (MEP). Thus, in this context Mll3 imparts a myeloid bias and is required for progenitor differentiation.

Within the HSPC compartment, Mx1-Cre-mediated Mll4 deletion increases overall LSK numbers that correspond to expanded LT-HSC, MEP, CMP and myeloid (CD11b+ Gr1+) cells, but not myeloid-biased HSCs, whilst reducing common lymphoid progenitors (CLP) and B-cell numbers [103]. Despite increased colony counts in successive rounds of myeloid CFU, competitive transplants using whole bone marrow or sorted LSK showed significantly reduced reconstitution ability as compared to wild-type mice. Non-competitive transplants allowed for comparable repopulation, suggesting that Mll4 mediates the response to stressed haematopoiesis [103]. Transcriptome profiling of *Mll4*^−/−^ LSK showed that Mll4 mediates the response to oxidative stress, and null cells were characterised by increased reactive oxygen species (ROS) and DNA damage confirming the functional defect. Interestingly, in HSPCs, Mll4 deletion prior to transformation or knockdown following retroviral MLL-AF9 transduction prevented leukaemogenesis. Transcriptional and functional testing of *Mll4*^−/−^ MLL-AF9 cells showed that increased ROS, DNA breakage and DNA-damage signalling was sufficient to promote myeloid differentiation. Thus, Mll4 protects HSPCs from DNA damage and oxidative stress, which allows for proper enforcement of a leukaemic differentiation blockade [103]. Abrogation of the tumour-suppressive DNA-damage response through deletion of genes such as *Brca1*, *Atm* and *Atr* are crucial to maintain the differentiation block caused by MLL-AF9.

In normal B-cells, Mll4 functions non-redundantly as an MLL/SET member that can positively influence all H3K4 methylation states, and is notably associated with H3K4me1 at enhancers and H3K4me3 at the promoters of genes that control B-cell signalling pathways [104,105]. Loss of Mll4 causes a proliferative advantage that expands the germinal centre (GC), which is enhanced by CD40 and IL4 stimulation and represents the cell type that is causative to diffuse large B-cell lymphomas (DLBCL) and follicular lymphomas (FL). Indeed, MLL4 loss of function mutations are especially prevalent in DLBCLs (30%) and FLs (90%) [106,107]. Most are monoallelic and less commonly biallelic, and generate a truncated MLL4 protein that is unable to methylate properly through its SET domain [105]. Mll4 deletion induced in forming GC B-cells (via Cγ1-Cre) or earlier B-cell progenitors (CD19-Cre) only results in significant expansion of the GC B-cell compartment when induced in the latter, and shows that Mll4 loss must be an early event that allows for the necessary chromatin and transcriptional remodelling events needed for transformation [105]. Cγ1-Cre-mediated Mll4 deletion combined with vav promoter-driven Bcl2 expression recapitulated the human disease phenotypes that occur in the progression from a FL to DLBCL. Altogether, this shows that MLL4 functions as a tumour suppressor in BLBCL and FL and constrains mature B-cell expansion.

## 6 SETD1A and SETD1B in haematopoiesis

Despite structural similarity, the mammalian orthologs of Drosophila SET1, SETD1A and SETD1B, fulfil distinct functions during embryogenesis [14]. Setd1a null embryos do not gastrulate following implantation and are unable to form ESCs [108]. Setd1b loss still permits the formation of the three germ layers, but severe growth retardation becomes apparent at E7.5 and embryos do not survive beyond E11.5 [108]. Correspondingly, only Setd1b loss is compatible with ESC proliferation. Interestingly, only Setd1a knockout is causative to a global loss of H3K4me1/2/3 in ESCs, suggesting that it is the major MLL/SET methyltransferase.

Conditional loss of Setd1a through Mx1-Cre in bone marrow cells caused a loss in B-cell numbers that corresponded to a block in differentiation from the pro-B to pre-B stage [109]. The block in B-cell development was correlated to losses in H3K4me3 at B-cell master regulators such as Pax5 and the IgH locus. Setd1a is also required for erythropoiesis [110]. Cre-mediated deletion driven by transgene expression from the erythropoietin promoter reduced the numbers of splenic erythroblasts (CD71^+^/Ter119^+^) and caused a mild anaemia. The differentiation defect was attributed to the loss of promoter H3K4me3 and transcription at erythroid lineage genes such as Klf1 and Gata1, which was caused by a loss of co-location of Usf1 at promoter loci, which may recruit Setd1a and cooperate to establish accessibility for gene expression. How these proteins locate their targets and cooperate in this context requires further investigation.

More recently, detailed analyses have demonstrated the impact of Setd1a and Setd1b deletion on HSPCs during foetal and adult haematopoiesis [111,112]. Vav-Cre-mediated Setd1a loss during foetal development leads to death 7 to 20 days post-birth from a significantly depleted LSK compartment, which must have failed to establish because of Setd1a deficiency in the first definitive HSCs that did not form or expand sufficiently [111]. Directed differentiation of ESCs to haematopoietic cells is often used as a model system for embryonic haematopoiesis in which detailed molecular mechanisms can more easily be dissected. Using this model system, it was demonstrated that a long intergenic non-coding RNA recruits both Setd1a and Mll1 to the promoters of the *Hoxb1–6* cluster, and enhances differentiation to Flk1^+^ mesoderm and subsequent differentiation to haemangiogenic and CD41^+^ c-Kit^+^ HSPCs [113]. In this study, Setd1a was the major contributor to HSPC differentiation as compared to Mll1. Also, Usf1 can recruit Setd1a to the *Hoxb4* promoter for H3K4me3 deposition which strongly enhances haematopoietic differentiation from ESCs [114]. Both studies showed that Mll1 does not affect *Hoxb4* expression, which may underlie the difference in ESC fate induction. In contrast, to the phenotype for Setd1a deletion, Vav-Cre-specific *Setd1b*^−/−^ null mice survive to a median of 25 weeks, suggesting that it is dispensable for the specification of a foetal haematopoietic system [112]. However, *Setd1b*^−/−^ null foetal HSPCs were not tested in any functional assays.

Two studies have explored the effect of Setd1a and Setd1b deletion on HSPC numbers during steady-state adult haematopoiesis, by transplanting recipient mice with HSPCs and subsequently knocking out the target gene following a latency period. Both ubiquitous Rosa26-CreERT2 and SCL-CreERT (HSC-specific) gene deletion had no effect on LT-HSC numbers [111]; however, Setd1a-deleted HSCs showed an inability to provide reconstitution in transplants and had reduced CFU output, confirming an intrinsic defect in these cells. Furthermore, ST-HSCs and MPPs are expanded, and significant depletion of downstream CMP and MEPs was seen. The loss of MEP is consistent with Setd1a regulating proper erythroid differentiation [110]. CLP and lymphoid-primed multipotent progenitor (LMPP) numbers or lymphoid potential in CFU following Setd1a depletion was not measured, leaving the question of Setd1a involvement in B-cell development unanswered [109]. Forced proliferation in secondary transplants with mixed bone marrow chimeras causes LT-HSC and broad HSPC depletion, showing that these cells become unable to compete with wild-type HSCs under replicative stress [111]. Given the significantly reduced LSK numbers in Vav-Cre *Setd1a*^−/−^ mice, this may be consistent with a failure to expand definitive HSCs as opposed to a failure to form HSCs entirely.

Similar to the eventually lethal Vav-Cre-induced adult phenotype, ubiquitous Setd1b deletion through Rosa26-Cre-ERT2 causes multilineage dysplasia, loss of normal bone marrow and splenic tissue architecture associated with thrombo- and lymphocytopenia, and varying levels of splenomegaly from an accumulation of extramedullary granulocytes and myeloid precursors. Transplantations of cells derived from both Setd1b knockout systems massively expanded the MPP compartment in the bone marrow, likely causing the accumulation of myeloid cells. Competitive transplantation of total bone marrow derived from *Setd1b*^−/−^ Rosa26-Cre-ERT2 showed no MPP expansion from null cells but the LT-HSC, LMPP, and LK compartments were significantly depleted and, correspondingly in the spleen, B-, T- and myeloid cell contribution from null cells was significantly reduced [112]. This negated a role for Setd1b in maintaining MPP numbers, and allowed for the demonstration of intrinsic Setd1b function that was masked by compensatory stress-based mechanisms that arise during defective haematopoiesis, imposed by a non-competitive setting.

Setd1b, in contrast, is required to maintain steady-state HSC and progenitor populations in the adult, but is not required for foetal haematopoiesis, highlighting the differences between foetal and adult blood. Transcriptionally, the inability to sustain HSPC homeostasis is associated with a loss of mitochondrial and metabolic associated processes indicative of reduced cellular activity. Indeed, plating of *Setd1b*^−/−^ c-Kit^+^ HSPCs in myeloid-supportive CFU showed a progressive reduction in output proportional to an increase in the age of *Setd1b*^−/−^ mice, suggestive of increasing stem cell exhaustion, and they are unable to sustain the proliferative demand imposed by MLL-ENL. Importantly, key markers across all haematopoietic lineages such as Mpo, Klf1 and Il7r were downregulated within HSPCs showing that Setd1b is required to maintain the expression of blood fate and lineage programming factors. Thus, Setd1b is intrinsically required to regulate HSPCs in the adult blood system, where it maintains proper myeloid and lymphoid differentiation.

Overall, both Setd1a and Setd1b are differentially required to maintain HSPC populations, although not all compartments were equally analysed (Table 2). Transcriptional analysis of bone marrow derived from Rosa26-CreERT2 *Setd1a*^−/−^ reconstituted chimeras showed that LT-HSC gene signatures, and DNA damage recognition and repair pathways were broadly downregulated [111]. Different functional assays demonstrated a ROS-independent reduction in DNA damage response and repair potential. H3K4me3 as shown by ChIP-qPCR was reduced at target genes such as *Fancd2* and *Orc5*, and in LSK, it is the major contributor to all forms of H3K4 methylation, indicating a direct role for H3K4 in mediating the knockout phenotype. This suggests that Setd1a functions to regulate LT-HSC identity and maintains genomic integrity especially in response to stressed haematopoiesis; however, whether this is direct transcriptional regulation and the role that H3K4 plays in mediating this response is unclear. In support of a role in directly regulating a transcriptional response, a SET-independent non-catalytic domain within SETD1A can bind Cyclin K, which maintains a DNA damage response through CDK12/13 to ensure the survival of MLL-AF9 AML cells [115]. Contrastingly, methylation mediated by SETD1A is required during replicative stress to protect replication forks by preventing RAD51 destabilisation, which protects nascent DNA from uncontrolled resection by the helicase/nuclease DNA-2 [116]. Ablation of H3K4 methylation allows for CHD4 localisation, which promotes fork degradation and subsequent genome instability. Indeed, the loss of SETD1A sensitises cells to genomic damage in line with what was observed in SETD1A null HSPCs, although no role could be implicated for Cyclin K in this process [111,115,116]. In addition, the ability of SETD1A-dependent methylation to control protein localisation and interaction has been shown to regulate the cellular localisation of YAP, which in turn promotes transcription factor association and downstream expression [117]. Therefore, SETD1A can utilise different functionalities in response to different contexts.

**Table 2:** MLL/SET family function in adult haematopoiesis.

	Adult haematopoiesis								
Gene	Effect on steady-state HSC numbers?	Effect on HSC function?	Effect on steady-state progenitor numbers?	Effect on progenitor function and expansion?	Required for HSPC expansion?	Required by MLL fusions or other blood diseases?	Methyltransferase activity required for haematopoiesis?	Effect on transcription	Role in differentiation and mature cells?
MLL1	Mx1-Cre - loss of LSK (severe cytopenia) [81] LSK CD48^neg -^ Failure to maintain quiescence [81] Vav-Cre (different allelic severities) 1) No change [79] 2) Significant LSK loss - no survival beyond 3 weeks [82]	Very low short term and no long term repopulation capacity [79,81,82]	Mx1-Cre – All progenitors lost (severe cytopenia) [81] Vav-Cre (different allelic severities) 1) no change [79] 2) Significant LSK loss – no survival beyond 3 weeks [82]	No progenitors generated [79,81,82]	Defective myeloid and lymphoid output in CFU across all HSPC populations [79,81,82] Sustains proliferation of myelo-erythroid progenitors [81]	SET functionality not required for MLL-AF9 transformation [86] Contributes to molecular pathogenesis [58,121,122] and can cooperate with MLL2 [123] MLL-AF9 can initiate and propagate absence of Mll1 [123,125]	No [86] haematopoiesis proceeds normally No change in global H3K4 [86]	Reduction in HoxA cluster (7, 8, 9) [78,81] and Mecom, Prm16, Hoxa9, Pbx1 and Eya1 [87]	Rag1-Cre - Required for pro-B to pre-B transition during B-cell development [83]
MLL2	Germline KO only – No indication for a requirement in haematopoiesis [88]	N/A	N/A	N/A	N/A	May be utilised instead of MLL1 for initiation and survival of adult MLL-AF9 AML [123,125]	Deletion reduces H3K4me2/3 at target genes in MLL-AF9 leukaemia [123]	N/A	Rosa-ERT2-Cre – Maintains Pigp expression, for cytokine signal transduction in macrophages [91]
MLL3	No MLL3 deletion or knockdown explored in adult HSPC [102]	N/A	N/A	N/A	N/A	Deleted in 7q AML [102]	N/A	N/A	N/A
MLL4	Mx1-Cre - LT-HSC elevated [103] However, increased frequency of symmetric division linked to attenuated self-renewal capacity [103]	LSK significantly reduced after competitive transplant [103] Repopulate in non-competitive setting [103]	LSK elevated in Mx1-Cre null mice [103]	LSK significantly reduced after competitive transplant [103] Null can repopulate in non-competitive setting [103]	Increased myeloid CFU output [103]	Participates in initiation and propagation of adult MLL-AF9 AML [103] Haploinsufficiency major cause of lymphomas [104,105]	In B-cells, global loss of mono-, di- and tri- H3K4 methylation on conditional deletion [105]	Deregulation of genes mediating response to oxidative stress, causing increased ROS and DNA damage signalling [103]	Required for normal germinal centre B-cell formation [105]
SETD1A	Rosa26-CreER^T2^/SCL-CreERT no change to LT-HSC numbers - following TAM induction post-transplant [111]	Rosa26-CreER^T2^/SCL-CreERT no donor contribution to myeloid + lymphoid cells [111] LT-HSC and ST-HSC remain but reduced in secondary transplants [111]	ST-HSC + MPP increased and CMP and MEP reduction - following TAM induction post-transplant [111]	R.26-CreER^T2^ MPP, CMP, MEP, GMP remain but reduced in secondary transplants [111]	Reduced myeloid CFU from R.26-CreER^T2^/SCL-CreERT [111]	No data	H3K4me1/2/3 depleted in knockout HSPCs [111] and H3K4me3 required at erythroid and B-cell lineage-specifying genes [109,110]	Deregulation of DNA damage response and repair, causing reduced genomic integrity [111]	Required for erythroid and B-cell differentiation [109,110] Not required in thymocytes [111]
SETD1B	Rosa26-Cre-ER^T2^ – Loss of LT-HSC contribution from null following TAM induction post-transplant [112]	Vav-Cre – LT-HSC numbers reduced after transplant [112] (non-competitive)	Rosa26-Cre-ER^T2^ – Loss of LMPP, LK but not MPP contribution from null following TAM induction post-transplant [112]	Vav-Cre – LMPP, LK reduced, MPP not affected (non-competitive) [112]	Adult Vav-cre-deleted mice show progressive exhaustion in myeloid CFU [112]	MLL-ENL transformed c-kit+ Vav-Cre KO cells exhaust by 4th round [112]	No SET mutant No ChIP in null HSPCs performed	Loss of haematopoietic TFs and genes involved in metabolic and mitochondrial processes [112]	No analysis

## 7 Requirement of MLL/SET proteins in MLL-rearranged leukaemia

Following MLL1 translocation, the C-terminal HMT SET domain is lost and the N-terminal DNA-binding and protein interaction (discussed above) domains become fused to a partner gene. Therefore, one wild-type MLL1 allele is lost in patient blasts. In rare circumstances, the second allele is also lost indicating a lack of selective pressure to retain it in leukaemia [[118], [119], [120]]. There are mixed reports on how MLL1 contributes to MLL-rearranged leukaemia. Initially, the prevailing thought was that MLL1 is always required [58,121,122]. Genetically deleting MLL1 through Cre and shRNA targeting of the C-terminal wild-type domain in primary murine or human MLL-AF9 cells impaired leukaemic proliferation and viability which was coupled to the loss of Ccna2 and Hoxa9 expression [121]. In mouse embryonic fibroblasts, this was attributed to MLL-AF9 not being able to bind the Hoxa9 locus in the absence of Mll1. Specifically, protein domain mutagenesis revealed a minimal set of recruitment requirements that are fulfilled by Mll1 through the DNA binding CXXC domain that must interact with the PAF1 elongation complex, and the PHD3 finger domain, which is lost in MLL1 fusions and recognises H3K4me2/3 [58]. Reintroduction of Mll1 into null cells rescued MLL-AF9 recruitment to Hoxa9 through pre-loading of Mll1 at the locus [58]. Small molecule inhibition of the MLL1 and WRD5 interaction crucial for MLL1 HMT activity impaired the growth of transformed primary murine and MLL-rearranged cell lines, but did not impair the proliferation of normal bone marrow progenitors in vitro [122]. This suggests that MLL1 enzymatic activity is required for the disease, which contrasts with previous reports that show that embryonic development, adult haematopoiesis and MLL-AF9 transformation can proceed in the absence of the SET domain [85,86]. Furthermore, conditional Mll1 deletion has demonstrated a need for Mll1 during adult haematopoiesis [79,81,82]. In further support, aberrantly increased expression of BCL6 correlates with increased MLL1 expression in both paediatric and adult MLL-rearranged B-cell acute lymphoblastic leukaemia (B-ALL) and defines a specific subset of patients with poor clinical outcome [84]. Specific to this subset of B-ALL is a dependency on BCL6, which can operate through a positive feedback loop with MLL1 to upregulate BCL6 (discussed above) and repress the proapoptotic BH3-only molecule BIM.

In opposition, the use of two independent conditional Mll1 knockouts and CRISPR-Cas9-mediated deletion showed that Mll1 loss did not significantly affect the self-renewal, proliferation and growth of MLL-AF9 leukaemic progenitors and generated a fatally transplantable disease with similar kinetics to control Mll1 heterozygote cells (which show no overt phenotype) [123]. However, unlike Mll1, inducible loss of Mll2 delays leukaemogenesis in vivo, and genetic deletion reduces leukaemic cell proliferation and viability. Interestingly, this effect was compounded when Mll1 and Mll2 were deleted together, indicating a synergistic contribution to leukaemic function. Transcriptionally, Mll2 directly targets *Magohb* and *Pigp*, which were downregulated following Mll2 loss, with and without simultaneous Mll1 deletion [88,91,124]. This combinatorial transcriptional response following Mll1/2 loss causes deregulation of NFκB, integrin β-3 and IL3 signalling, all major AML survival pathways and may represent critical vulnerabilities. Globally, Mll2 loss alone was the sole contributor to reductions in H3K4me2/3 at its targets, indicating that Mll2 SET function can drive leukaemic maintenance. Mechanistically, it is unclear how Mll1 cooperates with Mll2 from this study, and despite Mll2 not maintaining functional HSPCs during normal haematopoiesis, in MLL-rearranged leukaemia, the context allows for differential use of these proteins once transformation has occurred.

In HSPCs, Mll1 controls a specific transcriptional programme that is further upregulated and essential for MLL1 fusions and includes *Hoxa9*, *Meis1*, *Eya1*, *Mecom*, with the exception of *Prdm16* [87]. As opposed to deleting Mll1 following transformation, further work in the Ernst lab looked at whether Mll1 deletion would impair the ability of MLL-AF9 to initiate a leukaemia [125]. Mll1 null HSPCs were transformed by MLL-AF9 and displayed no differences in disease latency nor CFU proliferation and growth. This suggests that MLL-AF9 is able to induce the transcriptional programme needed for transformation that is lost when Mll1 is deleted. Only *Mecom* was not re-activated following MLL-AF9 induction, which suggests that MLL1 is required to prime the locus for MLL-AF9 [125]. Thus, the catalytic C-terminus of MLL1 that is not found in MLL1 fusions is not required for target gene activation in the context of transformation [85,86]. Therefore, MLL1 fusions can bypass MLL1 to activate genes for transformation, likely by aberrantly recruiting transcriptional activators such as the super elongation complex (SEC) [126].

The use of different inducible Mll1 alleles and the resulting differences in severities could be causative to the confounding results seen. Chen et al. make a robust case with different conditional strains to show that Mll1 is not required to maintain or initiate MLL-rearranged leukaemia. Even though MLL1 fusions can bypass MLL1, it does appear that MLL1 can prime loci (such as *Mecom* or *Hoxa9*) for subsequent activation by MLL1 fusions. However, the requirement for endogenous MLL1 usage as extrapolated from patient-derived data is compelling. As demonstrated for MLL-rearranged B-ALL, such usage may reflect on this particular subset of leukaemia and/or its human origin. Further extrapolation from patient-derived data and subsequent validation in relevant systems will be crucial to confirming how this is the case. It is unclear whether the catalytic domain of MLL1 is required in leukaemia. As MLL2 does not play a role as broad as MLL1 in haematopoiesis, MLL2 targets are a promising class for therapeutic investigation [88,91].

## 8 The impact of MLL1 fusions on haematopoietic development

Within infants (0–1 years), 80% of all B-ALL and 50% of AML cases are caused by MLL1 rearrangements [127]. These MLL1 fusion events occur in utero [[128], [129], [130]], and within each AML or ALL subclass, patients can be sub-categorised based on the fusion type. Interestingly, fusions such as MLL-AF9 can generate infant or paediatric B-ALL despite only causing AML in adults, and may be influenced by the foetal microenvironment, which imparts a lymphoid bias [127,131]. As age increases, MLL1 fusions become less prevalent as a subclass and increasingly require additional activating mutations, further highlighting the distinct nature of the infant disease [132,133]. Indeed, within infants, MLL1 rearrangements present with an effective 100% concordance rate between monochorionic twins, who rapidly develop overt leukaemia, suggesting that the fusion is sufficient for complete transformation [128,130,134].

Responsible for approximately 50% of infant B-ALL is the MLL-AF4 fusion. These patients are a particularly high-risk sub-group that retain very poor outcomes despite substantial improvements in paediatric ALL [135,136]. Patient blasts are arrested during early B-cell development at the pro-B stage (CD10 negative) and frequently co-express myeloid markers, suggesting that the transformation event is likely to occur in an early HSPC. This disease presents with some of the lowest somatic mutational landscapes of all sequenced cancers, they are clonal and lack significant heterogeneity with the exception of occasional activating mutations in RAS or FLT3, which are usually subclonal [133,137].

These patients can be further subcategorised by a lack of HOXA expression, which is linked to worse outcomes and increased relapse rates [138,139]. A HOXA signature correlates to significantly better outcomes and to expression of the reciprocal fusion AF4-MLL [137]. Specific to infant MLL-AF4 patients has been the detection of the fusion in bone marrow mesenchymal stromal cells [140]. This suggests that the fusion arises in a prehaematopoietic precursor and that its expression in non-blood cell types within the haematopoiesis-supportive stroma may shape the development of both the normal and leukemic haematopoietic compartments. This would impact the first definitive HSCs that emerge from a subset of endothelial cells known as the haemogenic endothelium, in a process termed endothelial-to-haematopoietic transition (EHT) (reviewed in [141]), and especially their further expansion and differentiation in the foetal liver.

To derive further insight into how MLL-AF4 may affect foetal haematopoiesis, MLL-AF4 has been expressed in cord blood (CB)-derived CD34+ cells and in ESCs. Indeed, viral induction of MLL-AF4 expression in human ESCs initially promotes the emergence of haematoendothelial precursors (CD31+ CD34+ CD45-) (HEPs), but then skews the balance of commitment towards endothelial fate, impairing haematopoietic output [142]. In this study, transformation was not achieved despite the expression of canonical MLL1 fusion oncodrivers HOXA9 and MEIS1, suggesting this is not the stage for transformation and that the in vitro ESC system is unable to recapitulate the foetal haematopoietic events during which transformation occurs. Upregulation of genes within the HOXA (9,13) and B (2,3,4,5,6) clusters was seen, and may play a role in regulating cell fate during the mesodermal to haematopoietic transition, which may have a functional impact on later leukaemia development. Furthermore, recent data from the Menendez lab using human pluripotent cells, showed that expression of both MLL-AF4 and its reciprocal fusion, AF4-MLL, strongly enhanced the specification of haemogenic- and endothelial-primed HEPs [143]. Thus, expression of AF4-MLL balances out the endothelial bias seen when MLL-AF4 is expressed alone, suggesting that both can cooperate during early blood specification to modulate both endothelial and haematopoietic cell fate. Significantly, 50% of infant MLL-AF4 patients express AF4-MLL, which is associated with activation of the HOXA cluster [137]. Molecularly, why this is the case is still an open question. The current thought is that AF4-MLL can disrupt transcriptional control, in part by interacting with the SEC through AF4, and create a more permissive environment to transcription, thereby increasing cellular plasticity and response to treatment. [[144], [145], [146]].

The use of CD34+ human CB has been more instructive with respect to leukaemic initiation. Initial attempts using human MLL-AF4 or in combination with activating mutations such as KRAS or FLT3, showed increased proliferative capacity and engraftment potential of transduced cells, but did not generate leukaemia [[147], [148], [149]]. Lin et al. showed that by replacing the human AF4 construct with the murine equivalent, a much higher retroviral titer was produced and suggested that human AF4 may have impeded previous studies [150]. Transduction of this hybrid in murine HSPCs generated an AML, whereas in human CB a pro-B ALL developed that expressed myeloid markers in xenografts. The pro-B phenotype lacked HOXA9 expression, which is consistent with a lack of AF4-MLL expression, and expressed RUNX1, which is correlated to poorer clinical outcome in adults [137,146]. Transduction of human CB with MLL-AF9 preferentially generated an AML and ALL; however, the differentiation arrest occurred at the later pre-B stage. This has been the most representative pro-B ALL model generated thus far for MLL-AF4. Accordingly, retroviral transduction and genetic editing of human CB with MLL-ENL and MLL-AF9 produces ALL, AML and mixed-lineage leukaemias with the latter fusion and entirely ALLs with MLL-ENL in xenografts, recapitulating the phenotypes of human disease for these two fusions [151,152].

Whilst there will be conservation in the mechanisms derived from CB or ESC towards the molecular processing that is directed when the fusion occurs in vivo, modelling how MLL fusions impact foetal haematopoiesis with relevant timing is essential to uncovering the characteristics of the infant disease. Indeed, foetal HSPCs possess distinct molecular characteristics that are the result of foetal-specific intrinsic and extrinsic factors, and the cell of origin for the disease is known to be a critical determinant towards the resulting pathology [131,144,[153], [154], [155], [156], [157], [158]]. Accordingly, identical knock-in models of adult MLL-AF9 and MLL-ENL leukaemia have shown that disease originates more efficiently from different HSPC populations when comparing both fusions, and that MLL-AF9 expression in LT-HSC causes a more aggressive leukaemia as compared to disease derived from GMP [157,159]. Important for infant leukaemia is to understand the contribution of developmental processes that shape the response to the leukaemic fusion, and may be especially important for MLL-AF4 which still displays dismal outcomes for patients. For example, the neonatal microenvironment can potentiate AMLs generated by retroviral transduction of murine HSPC with MLL-AF9 or MLL-ENL for mixed-lineage leukaemia output [131]. Furthermore, a MLL-ENL knock-in model showed that leukaemia initiates more efficiently from foetal and neonatal cells as compared to their adult counterparts, owing to younger HSPCs being more competent at activating the required oncogenic programme [160].

Barrett and Malouf et al. combined the use of a conditional MLL-AF4 invertor mouse with the Vav-Cre or VEC-Cre lines to target MLL-AF4 expression to the foetal blood system. The latter Cre strain targets the haemogenic endothelium, which is the precursor to all definitive blood [161]. Functional testing of total AGM and foetal liver-derived cells at E11, E12 and E14 in lymphoid CFU identified a window between E12-E14 that causes a significant increase in total B-lymphoid output (B220+ CD19+) relative to all other timepoints during blood development. The colonies were also visibly larger, suggesting an increased proliferative capacity relative to controls. E11 AGM-derived HSCs were unable to match control reconstitution levels in transplants further reinforcing this window. E12–14 HSCs showed the highest levels of repopulation, which were carried through to secondary transplants indicating enhanced self-renewal capacity through MLL-AF4. Indeed, at this stage the haematopoietic system undergoes substantial expansion and differentiation, which may provide the required context that MLL fusions can exploit during embryogenesis for leukaemia [162]. Sorting of HSC/MPP and LMPP revealed that it was the LMPP fraction that was the major contributor to B-lymphoid colony output, which had a pro-B phenotype, reflecting the developmental stage of B-cell arrest in ALL patients. Transcriptionally, MLL-AF4-expressing LMPP display higher levels of canonical MLL fusion oncoprotein targets such as *Meis1* and *Hoxa9*, B-cell lineage transcription factors *Ikaros* and *E2a,* and potential infant MLL-AF4 molecular drug targets *Hmga2* and *Lmo2* as compared to MLL-AF4-expressing HSC/MPP [163]. Wild-type LMPP innately express higher levels of B-cell lineage genes *Il7r* and *Pax5*, and higher levels of MLL-AF4 direct targets such as *Bcl2* and *Runx1* which are further upregulated in LMPP following MLL-AF4 induction. Thus, LMPP have the appropriate molecular characteristics to propagate Mll-AF4 pro-B ALL in this pre-leukaemic model, suggesting that it can act as the cell of origin for the disease. The observed B-lymphoid bias translated into a transplantable long-latency B-cell lymphoma not seen in infant patients, which have an acute nature. Despite this, this model allowed for the description of the necessary pre-leukaemic events needed to initiate an infant MLL-AF4 B-ALL and demonstrated how MLL-AF4 expression subverts normal haematopoietic development. Certainly, faithful models of B-ALL in mice have been difficult to generate, most likely due to species differences in blood function, which commonly manifest themselves through a myeloid bias and may be imposed by the murine microenvironment.

Recent work in human tissue has demonstrated the existence of a foetal-specific CD10 negative PreProB-progenitor that demarcates the first committed transition towards the B-cell lineage [164]. This progenitor only displays B-lymphoid and not myeloid output in vitro that is coupled with an upregulation of B-cell genes as it differentiates into downstream pro-B cells; however, it retains stem cell, myeloid and T-cell associated gene signatures, albeit at lower levels than multilineage upstream LMPP or myeloid progenitors. At the level of chromatin, PreProB cells retain accessibility at B-cell genes and in concordance with RNA expression, chromatin is accessible at foetal stem cell (*LIN28B*) and myeloid (*MPO*) genes. Interestingly, the PreProB pool undergoes substantial proliferation from 10 post-conceptional weeks (pcw), peaking at 11 in the foetal bone marrow and continues to be significantly high by 21 pcw despite downstream pro-B and B-cell progeny expansion. Thus, despite unilineage output in assays, this population appears to have a transitionary transcriptional and chromatin status. A high proliferative output in combination with a VDJ re-arrangement (D-JH) and transcriptional status that is similar to that of infant MLL-AF4 blasts, suggests that this CD10- PreProB progenitor could be a cell of origin candidate for the infant disease. It will be interesting to see how this committed progenitor responds to MLL-AF4, and whether innate priming with stem cell and myeloid genes (as seen with the LMPP) could be informative to any lineage switching mechanisms, which occur frequently at relapse [165,166].

## 9 Concluding remarks

It is increasingly clear that the MLL/SET family are essential to foetal, adult and diseased blood function and distinctly contribute to gene expression with cell type-specific behaviour. All commonly share a SET domain and have homologous architecture especially in their respective pairs, and yet can display distinct context-dependent functions. Thus far, specificity for each MLL/SET is not easily explained by the different ways that each protein can be recruited to chromatin, and it is likely that further factors play a part [14,167]. As MLL/SET proteins individually possess many domains and form multi-protein scaffolds for function, this is consistent with the notion that chromatin proteins locate their targets through the combinatorial action of multivalent interactions which come together to stabilise the final complex [14,58,68,168]. One aspect that may dictate MLL/SET usage is stoichiometric difference, which implies that their expression differs between developmental stages and tissue cell types [66].

Internally, small differences in domain structure are functionally important. For example, MLL2 cannot interact with LEDGF as it lacks a short series of 41 residues that are required to mediate a bridge between menin and LEDGF as seen with MLL1 (Fig. 2) [51]. Furthermore, these differences manifest as differing affinities towards the WRAD scaffold, which directly influence methylation capacity [35,36]. Pre-existing histone H2BK120ub1 is a feature of MLL/SET and COMPASS activation, which is also shared with the histone 3 lysine 79 methyltransferase DOT1L [36,41,169]. In MLL-rearranged leukaemia, aberrant recruitment of DOT1L by fusions also requires the action of the ubiquitination machinery for DOT1L to access chromatin, where it operates as an essential component of MLL fusion target gene activation through the deposition of its gene-activating H3K79 methylation mark [[170], [171], [172]]. Since DOT1L also plays a role in normal foetal and adult haematopoiesis, further structural and biochemical studies will be crucial to understand how all of these interactors and their cross-talk come to coordinate MLL/SET functional capacities in health and disease [169,[173], [174], [175]].

The role that these proteins play in coordinating cellular states in HSPCs is not always coupled directly to methylation action. For example, haematopoiesis and MLL-rearranged leukaemogenesis can proceed normally in the absence of the Mll1 SET domain, where Mll1 recruits MOF for H4K16ac at target genes [54,86]. Interestingly, Mll4 also co-locates with MOF and H4K16ac at a subset of its target sites [176]. Mechanistically, Mll4 can recognise H4K16ac via its PHD6 finger domain, and Mll3 via PHD7. As discussed above, SETD1A can function in distinct contexts to directly recruit co-factors, or through methylation, mediate protein access and activity with functional consequences. Further work that explores domain functionality in haematopoietic contexts will crystallise how much functionality is conserved across the MLL/SET family and when HMT activity is applicable.

MLL/SET can control key HSPC programmes such as self-renewal for HSC function (MLL1), the insurance of genomic integrity in proliferating HSCs (SETD1A), mediation of differentiation from HSC to downstream progenitors (MLL3) (Table 1, Table 2). As discussed above, MLL1 fusions are able to skew the balance between haematopoietic and endothelial cell fate commitment, and both Setd1a and Mll1 have been shown to play in role in haematopoietic specification from ESC [113,114,177]. How MLL/SET proteins function during the establishment of the haemogenic endothelium and/or early primitive haematopoietic progenitors remains underexplored (Table 1). Given the multi-domain nature of MLL/SET and that foetal blood cells are distinct from their adult counterparts, it is entirely possible that new molecular mechanisms could be found [154,156]. As noted in Table 1, there is much to learn about the developmental phenotypes of the MLL/SET family.

## Author statement

Eric Antunes: Writing - Original draft preparation. Katrin Ottersbach: Writing - Reviewing and Editing.

## Declaration of competing interest

The authors have no conflicts of interest to declare.
